# Role of endothelial nitric oxide synthase VNTR (intron 4 a/b) polymorphism on the progression of renal disease in autosomal dominant polycystic kidney disease

**DOI:** 10.12861/jrip.2014.21

**Published:** 2014-07-01

**Authors:** Ramprasad Elumalai, Soundararajan Periasamy, Gnanasambandan Ramanathan, Bhaskar VKS Lakkakula

**Affiliations:** ^1^Department of Nephrology, Sri Ramachandra Medical College and Hospital, Sri Ramachandra University, Chennai, India; ^2^Department of Biomedical Sciences, Sri Ramachandra University, Chennai, India; ^3^Sickle Cell Institute Chhattisgarh, Raipur, India

**Keywords:** *NOS3* gene, Autosomal dominant polycystic kidney disease, Chronic kidney disease

## Abstract

**Introduction:** Autosomal dominant polycystic kidney disease (ADPKD) is an inherited disorder, and it is mainly associated with renal cyst formation. Several studies have also shown that these mutations regulate the physiology of epithelial tissues and determine renal cyst formation and growth in polycystic kidney disease (PKD). Nitric oxide (NO) is also considered to be an important factor involved in the deterioration of renal function.

**Objectives:** The aim of the current study is to determine the frequency of *NOS3* 27-bp VNTR in ADPKD patients and to investigate the role of *NOS3* 27-bp VNTR genotypes in the modification of progression of renal disease in ADPKD.Patients and Methods: The hypothesis was investigated by studying the South Indian population of 53 ADPKD patients and 94 unrelated healthy controls. The genotyping was performed by polymerase chain reaction and electrophoresis. Genotypes were compared between ADPKD and controls using the χ^2^-test. Univariate and multivariate logistic regression analyses were performed to assess the effect of genotypes and hypertension on the progress of chronic kidney disease (CKD). A stratified analysis was also performed to assess the evidence of the modification of hypertension-CKD relationship among VNTR genotypes.

**Results:** The *NOS3* 4a allele frequencies were 21.3% and 13.2% respectively for controls and ADPKD groups. The *NOS3* VNTR genotypes and alleles were not associated with ADPKD. The univariate analysis showed that age, hypertension and *NOS3* VNTR influenced the advancement of CKD.

**Conclusion:** The present study confirms the significant association between the 27-bp VNTR and CKD advancement among the ADPKD patients in the South Indian population.

**Implication for health policy/practice/research/medical education:**

In the present study, nitric oxide synthase (NOS 3) gene 27-bp VNTR polymorphism was investigated in autosomal dominant polycystic kidney disease (ADPKD) and healthy subjects. This study demonstrated that the distribution of NOS3 VNTR genotypes in ADPKD did not differ from those in controls. But the influence of NOS3 VNTR on the progression of chronic kidney disease (CKD) in ADPKD was observed. NOS3 VNTR 4a allele might be a valuable indicator in the prediction of progress of CKD in south Indian populations.

## Introduction

Autosomal dominant polycystic kidney disease (ADPKD) is an inherited disorder of worldwide origin, caused by mutations of polycystic kidney disease (PKD) genes, PKD1 or PKD2 (1). It is predominantly associated with renal cyst formation. Several studies have also shown that these mutations regulate the physiology of epithelial tissues and determine the renal cyst formation and growth in PKD (1,2). Several lines of evidence suggest that the endothelial dysfunction, secondary to an impaired release of nitric oxide (NO), exists in ADPKD (3). NO is the molecular counterpart of the endothelium-derived relaxing factor and a key signaling molecule that controls blood pressure and nerve impulses (4-6). NO is synthesized by the endothelial NO synthase (eNOS) through the conversion of L-arginine to L-citrulline, using molecular oxygen. The gene coding for eNOS (*NOS3*) is located on 7q36 (7). ADPKD is associated with altered endothelial-dependent vasodilation and decreased vascular production of NO. This has led to the hypothesis that it might be a modifier gene in ADPKD (8-10). Several single nucleotide polymorphisms (SNPs) have also been described in the *NOS3* gene and some of them have been associated with altered eNOS function, leading to impaired NO synthesis. A missense variant in exon 7 (Glu298Asp) and 27-base pair (bp) variable number of tandem repeat (VNTR) in the intron-4 of *NOS3* gene are known to alter eNOS expression (11,12). The relationship between *NOS3* gene polymorphisms and some diseases have already been reported (13-16). Further, reports have also shown that eNOS (*NOS3*) 27-bp VNTR polymorphism might have a modifier effect on the ADPKD progression (11). Nevertheless, there is no data available with reference to the *NOS3* gene polymorphisms and their association with ADPKD or its progression in the Indian population.

## Objectives

The objectives of the current study were to determine the frequency of *NOS3* 27-bp VNTR in ADPKD patients and to further investigate the modification of renal disease progression in ADPKD by the *NOS3* 27-bp VNTR genotypes.

## Patients and Methods

### Study population

The study population comprised of a total of 147 individuals (53 ADPKD and 94 controls), who were selected from the Department of Nephrology, Sri Ramachandra University, Chennai, India.

### Laboratory study

Hematological, serum biochemical and electrolyte indices were measured for all the study subjects. Estimated glomerular filtration rate (eGFR) was assessed based on Modification of Diet in Renal Disease (MDRD) formula (17). The total number of cysts in the ADPKD patients was detected by using ultrasound imaging. Only those ADPKD patients, who fulfilled the standard diagnostic criteria, were included in the study (18). Further, the stage of chronic kidney disease (CKD) of the study subjects was determined using eGFR and all the ADPKD patients were then divided into two groups; early (CKD stages 1-3) and advanced stages (CKD stages 4 and 5). Individuals without diabetes, hypertension and kidney related diseases were considered as controls. Three mL of blood samples were collected from all the participants and DNA was extracted from whole blood by the Phenol chloroform extraction and ethanol precipitation techniques (19). Genotyping for *NOS3* 27-bp VNTR polymorphism was performed by PCR-electrophoresis methods (20). The amplicons were examined by agarose gel electrophoresis. The PCR amplicon’s size of 393 bps corresponded to 4a/a homozygotes, 420 bps to 4b/b homozygotes and 393 and 420 bps to 4a/b hetrozygotes.

### Ethical issues

The study was approved by the Institutional Ethics Committee of the Sri Ramachandra University, Chennai, India. A written informed consent was obtained from all the study participants.

### Statistical analysis

Allele frequencies were determined by direct counting of alleles at each locus. The genotype distribution was evaluated for Hardy–Weinberg’s equilibrium (HWE). The association between *NOS3* 27-bp VNTR polymorphisms and ADPKD was analyzed using χ^2^-test. Odds ratios and 95% confidence intervals (CIs) were calculated. Univariate and multivariate logistic regression analyses were performed to assess the effect of genotypes and hypertension on CKD progression in ADPKD. Mantel-Haenszel stratified analysis was also performed to assess the modifying effects of *NOS3* 27-bp VNTR. All statistical analyses were performed with SPSS statistical software version 16.0 (SPSS Inc, Chicago, Illinois) for Microsoft Windows.

## Results

Participants of the study comprised of 94 controls and 53 patients with ADPKD. The mean ages of the study participants were 52.1±12.2 years for the control group and 49.20±10.1 years for ADPKD cases. The *NOS3* 27-bp VNTR genotypes are following the HWE in control group as well as in ADPKD group (Table 1). The *NOS3* VNTR mutation was found in 36 (38.3%) controls and 12 (22.6%) ADPKD cases. The *NOS3* 4a allele frequencies were 21.3% and 13.2% respectively for controls and ADPKD cases. The *NOS3* genotypes were not significantly associated with ADPKD (p= 0.136). The *NOS3* VNTR did not show a significant association with ADPKD at allele level (4b vs. 4a: OR= 0.563, 95% CI= 0.290–0.109, p= 0.086) (Table 1). Among ADPKD, 37 (69.8%) cases showed advanced CKD stages with mean age of 51.6±10.7 yrs and 16 cases (30.2%) showed early CKD stages with 39.8 ± 11.7 yrs of age. The *NOS3* VNTR was found to be associated with mutant allele and was observed only in the advanced CKD stage group (Table 2). The univariate analysis revealed that the age and hypertension had contributed to the significant advancement of CKD, while the gender and family history of diabetes had no effect on CKD advancement (Table 2). After controlling for the other variables in multivariate analysis, only *NOS3* VNTR mutant genotypes and age were found to significantly increase the risk of CKD advancement in ADPKD (Table 2). Mantel-Haenszel statistics test showed no evidence for heterogeneity of the effect of hypertension on CKD stages (Table 3).

**Table 1:** Results of association tests between *NOS3* 27-bp VNTR polymorphism and ADPKD patients in different models

**Genotype**	**Controls n (%)**	**ADPKD n (%)**	**OR (95%CI)**	**P value**
4bb	58 (61.7)	41 (77.3)	Reference	
4ab	32 (34)	10 (18.9)	0.442 (0.195-0.999)	0.136*
4aa	4 (4.3)	2 (3.8)	0.707 (0.124-4.045)	
4ab+4aa	36 (38.3)	12 (22.7)	0.472 (0.219-1.014)	0.052
4b	148 (78.7)	92 (86.8)	Reference	
4a	40 (21.3)	14 (13.2)	0.563 (0.290-0.109)	0.086
HWp	0.875	0.197		

4b: *NOS3* VNTR wild type allele; 4a: *NOS3* VNTR mutant allele; HWp: Hardy-Weinberg equilibrium p value. *degrees of freedom (d.f)=2

**Table 2:** Characteristics of ADPKD cases and their association with chronic kidney disease stage.

**Gene**	**Genotype**	CKD Stages		Univariate		**Adjusted effects of risk factors**	
		**Early stage (%)**	**Advanced stage (%)**	**OR (95% CI)**	**P value (χ** ^2^ **)**	**OR (95% CI)**	**Wald's test** **p value**
*NOS3* VNTR	4bb	16 (100)	25(67.6)	1	0.035	1	
	4ab	0 (0)	10 (27.0)	-		-	-
	4aa	0 (0)	2 (5.4)	*-*		*-*	*-*
Age	≤40	10 (62.5)	7(18.9)	1	0.007	1	0.031
	(40-60]	5 (31.3)	22 (59.5)	6.29 (1.60,24.73)		6.59 (1.19,36.60)	
	>60	1 (6.3)	8 (21.6)	11.43 (1.16,113.16)		-		
Sex	F	7 (26.9)	17 (45.9)	1	0.562	1	
	M	19 (73.1)	20(54.1)	1.09 (0.34,3.56)		1.55 (0.28,8.67)	0.619
HT	No	6 (37.5)	1 (2.7)	1	0.002	1	
	Yes	10 (62.5)	36 (97.3)	21.6 (2.32,200.86)		21.6 (2.32,200.86)	0.001
FH- DM	No	2 (12.5)	10 (27.0)	1	0.215	1	
	Yes	14 (87.5)	27 (73.0)	3.86 (0.074,2.01)		0.547 (0.075,3.99)	0.552

FH-DM: family history of diabetes mellitus

**Table 3:** Association between CKD stages and hypertension stratified by *NOS3* VNTR genotypes.

**Gene**	**Genotype**	**OR(95% CI) for HT**	**Homogeneity test P-value**
*NOS3* VNTR	4bb	14.4 (1.53,135.52)	NA
	4ab	-	
	4aa	-	
M-H combined		14.4 (1.53,135.52)	

## Discussion

In view of the strong association of ADPKD with hypertension, the eNOS enzyme responsible for NO production and variations in its gene expression could be linked to hypertension. In this context, *NOS3* gene has long been thought of as a candidate gene for ADPKD. In this study, after investigating 94 controls and 53 ADPKD subjects, no significant association was observed between the genotypes and alleles of *NOS3* 27-bp and ADPKD. The univariate analysis revealed that the *NOS3* VNTR, age and hypertension contributed to the advancement of CKD. After controlling other variables in multivariate analysis, only VNTR genotypes and age were found to increase the risk of CKD advancement in ADPKD. Stratified analysis further showed that the *NOS3* VNTR was an effect modifier of the relationship between hypertension and CKD advancement among the ADPKD patients. The results were also consistent with the existing reports in this regard. The first study linking this VNTR and hypertension was published by Miyamoto and co-workers (21). Though these findings were not substantiated in the Australian population (22), a positive association was observed between the plasma nitrite or nitrate levels and blood pressure in normotensive African Americans (23). Further, the a/a genotype of the *NOS3* VNTR was shown to significantly increase the risk of developing hypertension in Indian men (24). Following the conflicting results generated from these association studies, Intron 4 VNTR and haplotype analysis of several other SNPs of *NOS3* showed significant association with daytime systolic blood pressure (25). In the present study 4a allele was significantly associated with CKD advancement in ADPKD. Higher incidence of 4a allele was observed among Czech patients with end-stage renal disease (ESRD) caused by ADPKD (26). On the contrary, *NOS3* VNTR polymorphism showed no effect on the age at ESRD in unrelated ADPKD patients from Belgium and the north of France (11). Several studies have demonstrated that a decrease of NO synthesis and release may be important in the progression of renal disease (27), and a significant endothelial dysfunction has also been documented in ADPKD patients (28). Direct analysis of *NOS3* gene polymorphisms in ADPKD patients have also revealed inconclusive results from many populations. Although no direct association between *NOS3* VNTR and ADPKD has been observed, patients who carried 4a allele showed faster ESRD progression in the group of ADPKD (29). Higher frequency of the *NOS3* 4a allele carriers among CKD children suggests that the *NOS3* VNTR may be associated with an increased risk of chronic renal failure (30).

## Conclusion

In conclusion, we identified the significant association between *NOS3* 27-bp VNTR and CKD progression in ADPKD. However, more focused research in this regard is required to further validate our findings.

## Acknowledgements

The authors would like to thank Sri Ramachandra University for providing necessary facilities and the subjects.

## Authors’ contributions

RE, SP and BLVKS conceived the study and contributed reagents and tools. RE, GR and BLVKS performed the experiments. RE and BLVKS analyzed the data and drafted the final manuscript; all authors read, revised, and approved the final manuscript.

## Conflict of interests

The authors declared no competing interests.

## Ethical considerations

Ethical issues (including plagiarism, data fabrication, double publication) have been completely observed by the author.

## Funding/Support

No funding from any source.
